# Glucocorticoid-Mediated Developmental Programming of Vertebrate Stress Responsivity

**DOI:** 10.3389/fphys.2021.812195

**Published:** 2021-12-21

**Authors:** Ian M. Gans, James A. Coffman

**Affiliations:** ^1^MDI Biological Laboratory, Salisbury Cove, ME, United States; ^2^Graduate School of Biomedical Science and Engineering, University of Maine, Orono, ME, United States

**Keywords:** cortisol, glucocorticoid receptor, adaptive, dynamics, allostasis, early life stress, gene regulation, Klf9

## Abstract

Glucocorticoids, vertebrate steroid hormones produced by cells of the adrenal cortex or interrenal tissue, function dynamically to maintain homeostasis under constantly changing and occasionally stressful environmental conditions. They do so by binding and thereby activating nuclear receptor transcription factors, the Glucocorticoid and Mineralocorticoid Receptors (MR and GR, respectively). The GR, by virtue of its lower affinity for endogenous glucocorticoids (cortisol or corticosterone), is primarily responsible for transducing the dynamic signals conveyed by circadian and ultradian glucocorticoid oscillations as well as transient pulses produced in response to acute stress. These dynamics are important determinants of stress responsivity, and at the systemic level are produced by feedforward and feedback signaling along the hypothalamus-pituitary–adrenal/interrenal axis. Within receiving cells, GR signaling dynamics are controlled by the GR target gene and negative feedback regulator *fkpb5*. Chronic stress can alter signaling dynamics *via* imperfect physiological adaptation that changes systemic and/or cellular set points, resulting in chronically elevated cortisol levels and increased allostatic load, which undermines health and promotes development of disease. When this occurs during early development it can “program” the responsivity of the stress system, with persistent effects on allostatic load and disease susceptibility. An important question concerns the glucocorticoid-responsive gene regulatory network that contributes to such programming. Recent studies show that *klf9*, a ubiquitously expressed GR target gene that encodes a Krüppel-like transcription factor important for metabolic plasticity and neuronal differentiation, is a feedforward regulator of GR signaling impacting cellular glucocorticoid responsivity, suggesting that it may be a critical node in that regulatory network.

## Introduction

Glucocorticoids (GCs), steroid hormones produced by the vertebrate adrenal cortex (and hence also known as *cortico*steroids), function to maintain homeostasis in a changing environment by dynamically regulating diverse aspects of physiology, including metabolism and immunity. The role of GCs in human health was first glimpsed in the late 19th century when Thomas Addison found that adrenal extract had therapeutic properties for patients with a multi-symptom wasting disease (now known as Addison’s Disease or adrenal insufficiency; Timmermans et al., 2019). Decades later in the early-mid 20th century, it was noted by Philip Hench that temporary remissions of rheumatoid arthritis were associated with conditions that stimulate the adrenal cortex, including pregnancy (a condition now known to increase maternal GC levels several-fold). In 1946, four adrenal products were purified by Edward Kendall, and Compound E, now known as cortisol, was found by Hench to have remarkable benefits for patients with severe arthritis (Hench et al., 1949). This led to the award of the Nobel Prize in Physiology and Medicine in 1950, and today synthetic GC analogs of cortisol are widely prescribed for their anti-inflammatory properties.

Cortisol is commonly referred to as a (or inaccurately as “*the*”) stress hormone and is one of the key hormonal regulators of the physiological response to stress, along with adrenaline and noradrenaline. In response to stress, these hormones orchestrate usage of energy stores, and prime the body to respond to threat. In contrast to the physiological effects of adrenaline which occur almost instantaneously, the effects of GCs largely involve changes in gene transcription and thus require several minutes to occur. More rapid, non-genomic mechanisms of GC action also exist, however these are less well understood (Panettieri et al., 2019). This review focuses primarily on the effects that GCs exert through regulation of gene expression.

**Figure 1:**
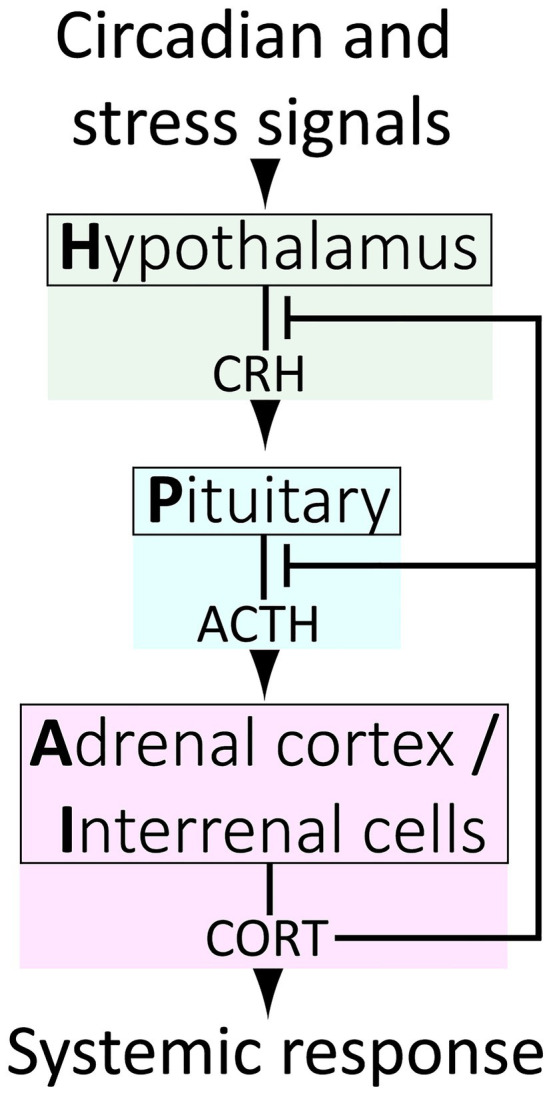
The vertebrate Hypothalamus-Pituitary–Adrenal/Interrenal (HPA/I) axis.

The release of endogenous GCs (primarily cortisol in primates and fish, corticosterone in amphibians, reptiles, birds and rodents; referred to hereafter as CORT in either case) into the bloodstream is result of a hormone cascade that occurs upon activation of the Hypothalamus-Pituitary–Adrenal (HPA) neuroendocrine axis (Figure 1). HPA activation in response to signals from the central nervous system cause corticotrophin releasing hormone (CRH) to be released by the hypothalamus into the pituitary portal circulation. CRH then acts on the anterior pituitary gland causing the release of adrenocorticotropic hormone (ACTH) which travels through the blood stream to the adrenal glands triggering the release of CORT into the blood.

**Figure 2:**
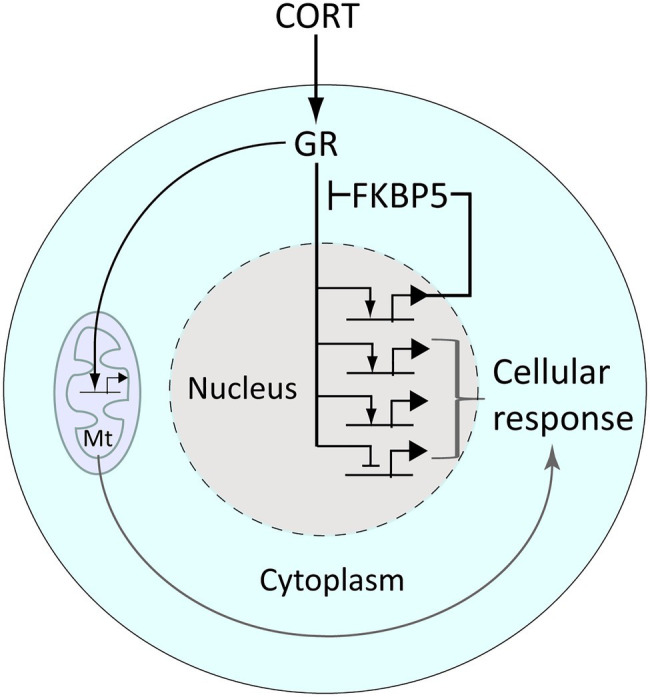
The glucocorticoid receptor (GR) regulates gene expression and the cellular response to glucocorticoid signaling. CORT diffuses freely into cells wherein it binds the GR, releasing it from a chaperone complex (which includes the protein Fkbp5) that anchors it in the cytoplasm. CORT-activated GR translocates into the nucleus, wherein it regulates the transcription of genes bearing glucocorticoid-response elements (which include *fkbp5*) or *via* tethering to other transcription factors. Activated GR also enters and regulates gene activity within mitochondria (Mt). These gene regulatory effects combine to produce a cellular response to CORT, for example, metabolic remodeling.

As small lipophilic molecules, GCs freely diffuse from the blood stream through cell membranes and into the cytoplasm of cells. Once inside cells GCs bind and activate cognate receptors, the glucocorticoid receptor (GR, encoded by the *NR3C1* gene) and the closely related mineralocorticoid receptor (MR, encoded by *NR3C2*). These receptors are protein transcription factors that upon GC binding translocate to the nucleus to regulate gene transcription, which includes GR-mediated activation of the GR-antagonist *FKBP5*, providing intracellular negative feedback regulation (Figure 2). The changes in gene expression mediated by the GR result in metabolic remodeling and other aspects of cell physiology that comprise the cellular response to GCs (Figure 2). In addition, the GR is known to translocate into mitochondria and regulate mitochondrial gene expression, further contributing to the cellular response to GCs (reviewed by Kokkinopoulou and Moutsatsou, 2021; Figure 2).

Both the GR and MR have a central DNA binding domain flanked by an N-terminal domain and a C-terminal ligand-binding domain. The high affinity of the MR for GCs (as well as other steroid hormones such as aldosterone) leads to its activation at even low physiological levels of GC hormone (Reul and De Kloet, 1985; Gomez-Sanchez and Gomez-Sanchez, 2014). In comparison, the GR has a nearly 10-fold lower binding affinity for CORT, and its activation is thus sensitive to fluctuations in CORT levels that occur with circadian (roughly 24-h) rhythmicity and on more rapid timescales (within minutes in response to acute stressors). Because the GR is expressed in nearly all cell types and binds thousands of genomic sites in a given target tissue (Sacta et al., 2016), changes in circulating CORT levels produce broad changes in gene expression, physiology and behavior, and are a primary means of coordinating an adaptive organism-level response to stress and/or a changing environment.

Within the cell nucleus, regulation of gene expression by the GR is accomplished in part through its direct binding to glucocorticoid response elements (GRE) in regulatory DNA regions. In the canonical pathway, nuclear GR dimers interact with palindromic GRE sites. However, binding of GR monomers to half sites, inverted GRE (half sites occurring on opposite strands) and indirect “tethered” interactions with DNA mediated by other transcription factors, also occur (reviewed thoroughly by Weikum et al., 2017). The regulation of transcription by the GR involves recruitment of numerous cofactors, can be either activating or repressive, and is highly context dependent. The GR is also subject to various post-translational modifications (e.g., acetylation, phosphorylation, sumoylation, ubiquitylation, nitrosylation) that further control its activity. Thus, although the GR is among the most well-studied transcription factors, the various modes and contextual determinants of its function continue to be fertile ground for research.

## The Functional Significance of Glucocorticoid Signaling Dynamics

**Figure 3:**
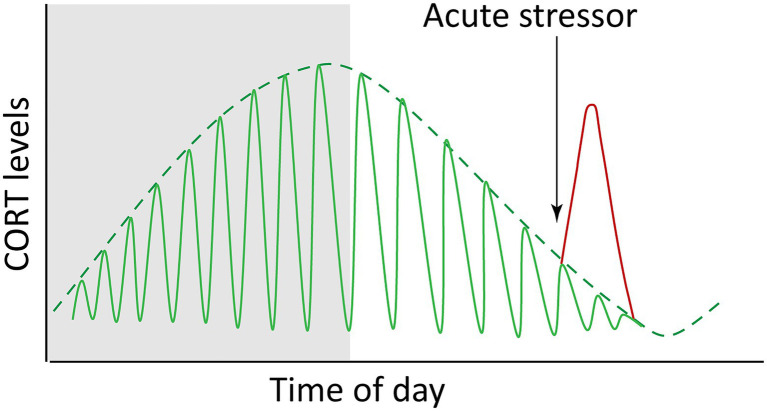
Endogenous glucocorticoids (cortisol and corticosterone, CORT) fluctuate both with circadian and ultradian rhythms (green lines) and in response to acute stress (red line). The idealized graph depicts the situation in a diurnal animal (i.e., with a waking phase during daylight hours; nighttime is depicted by shading). The circadian dynamics are reversed in nocturnal animals.

The HPA axis is a highly dynamic and responsive system, so to understand how it maintains both homeostasis and stress responsiveness, we must consider its temporal regulation. It is widely appreciated that at the systemic level, negative feedback exists between HPA tissues, wherein circulating GCs act upon the hypothalamus and pituitary to downregulate further CRH, ACTH and GC release (Figure 1). However, HPA axis regulation is more complex, and our understanding remains incomplete. At the top level, the hypothalamus receives inputs from complex neural circuits involving the hippocampus, amygdala and prefrontal cortex, which regulate CRH release, and each of these brain regions exert different and context-dependent controls. For example, the hippocampus and amygdala, respectively, exert primarily inhibitory and facilitative influences on hypothalamic CRH production (Jankord and Herman, 2008). In more granular detail, different loci within a brain region may govern responses to different modes of stimuli. For example, the central and medial nuclei of the amygdala separately facilitate HPA responses to systemic (e.g., inflammation) or psychogenic (e.g., restraint) stress. It is also of interest to note that CRH is a neuropeptide produced throughout the brain, and that hypothalamic neurons are the only population which respond to GCs by downregulating CRH (Watts and Sanchez-Watts, 1995; Watts, 2005). In contrast, CRH production is strongly stimulated by GCs in the amygdala in a manner inverse to the repression by GCs on hypothalamic CRH. The amygdala also mediates aspects of GC-induced anxiety and fear, and it may activate the hypothalamus through interruption of basal inhibition (Hakamata et al., 2017; Kinner et al., 2018). In all, regulation of the HPA axis *via* integration of complex circuits almost certainly provides a survival advantage by conferring adaptive plasticity. However, this distributed regulatory framework also appears to allow for overriding of the negative feedback that controls normal dynamics. Under normal conditions, these dynamics involve both regular fluctuations on circadian and ultradian timescales and transient responses to acute stressors (Figure 3), but these dynamics can be altered in unhealthy scenarios. For instance chronic stress can remodel brain regions, shrinking dendrites in the hippocampus while expanding them in the amygdala (Lakshminarasimhan and Chattarji, 2012; McEwen et al., 2016), potentially altering the ratio of inhibitory/activating inputs on the hypothalamus and affecting HPA dynamics.

### Circadian Dynamics

Within the hypothalamus, the suprachiasmatic nucleus (SCN) is the well-established site of the central circadian clock, sitting above the optic chiasma and receiving input directly from light-sensitive neurons. Nearby, the paraventricular nucleus (PVN) is the source of hypothalamic CRH secretion and receives inputs from the SCN, generating light-dependent activation of the HPA axis and circadian oscillations in GC production (Kalsbeek et al., 2012). Glucocorticoids thus relay information from the central or “master” circadian pacemaker to so-called “slave” clocks in additional tissues to coordinate organism-wide timing, with daily peaks in GC levels preceding the active behavioral phase (early morning in humans and zebrafish, evening in rodents). Various and layered mechanisms coordinate with circadian levels of GCs to generate robust rhythmicity in GC-signaling. Examples include downregulation of GR protein activity through post-translational acetylation by the core circadian protein CLOCK (Circadian Locomoter Output Cycles Kaput, a transcription factor and acetyltransferase; Nader et al., 2009), as well as diurnal rhythmicity in levels of GC transport proteins that regulate the availability of free cortisol in the blood stream (Debono et al., 2016).

Most cells possess autonomous 24-h clocks, but phase coherency at the tissue or whole-organism level requires synchronization by circadian signals, many of which (including GC rhythmicity) originate from the SCN (Schibler et al., 2015). Strikingly, a short pulse of the synthetic GC dexamethasone (DEX) is sufficient to synchronize circadian gene expression in cell cultures, while injections of DEX are able to shift or reset the phase of circadian gene expression in peripheral tissue of live mice based on the timing of the injection (Balsalobre et al., 2000). This ability of GCs to alter the circadian phase does not apply to cells of the SCN, however, where the notable lack of GR expression likely protects the central clock from the influence of acute hormone spikes.

In zebrafish, tonic concentrations of DEX have been shown sufficient to rescue 24-h cell-cycle rhythms in larvae lacking corticotrope cells, suggesting that the presence of GCs above a threshold concentration is sufficient for synchronization of some peripheral clocks, and that rhythmicity of GC levels may be dispensable for some processes (Dickmeis et al., 2007). The cell cycle is not the only rhythmic biological process, however, and it’s possible that while peak GCs entrain cell-cycle, trough periods may provide rhythmic impetus for other processes (especially considering that the GR may act as either an activator or repressor of target gene transcription). Diurnal lulls in GC levels could also free up a cell’s signaling bandwidth for response to additional or subsequent signals, including providing slack in the system necessary for HPA re-activation, such as in a “fight-or-flight” scenario. Dynamic flexibility allows for additional GC peaks to be superimposed on the 24-h light-driven oscillations as may be warranted by altered feeding habits, perceived threats, or other conditions (Figure 3). For example, robust feeding-induced GC rhythms depend on an adrenal clock which normally reinforces SCN clock timing but can become uncoupled from it and generate independent hormone dynamics (Chung et al., 2017).

### Ultradian Dynamics

The circadian profile of circulating CORT is not achieved through smooth continuous release, but rather is driven by pulsatile release of hormone occurring on the order of hourly, or in other words with ultradian frequency (Veldhuis et al., 1989; Jasper and Engeland, 1991; Stavreva et al., 2009). While the frequency of these pulses remains relatively constant, varying amplitude drives circadian GC level (Figure 3), with the highest pulses occurring just prior to the waking phase. It was once thought that an ultradian pacemaker must reside in the brain, however studies in the last decade by the Lightman laboratory using automated hormone infusions and measurements in rats have demonstrated these pulsatile characteristics result from the combination of feedback and feedforward signals and delays among HPA tissues (Walker et al., 2010, 2012; Spiga et al., 2011). As predicted by initial computational modeling, pulsatile infusions of ACTH were found to induce pulsatile corticosterone release in rats whose endogenous ACTH production was suppressed by the synthetic GC methylprednisolone. This was followed by pulsatile transcription of genes involved in steroidogenesis, similar to gene pulsing effects previously seen *in vitro* with cycles of GR activation and association with chromatin leading to ultradian pulses of gene transcription (Stavreva et al., 2009). Interestingly, constant infusion of ACTH caused no induction of CORT, indicating that the pulsatile nature of ACTH signal is key to normal adrenal function. In contrast, constant infusion of CRH was found sufficient to drive pulsatile release of both ACTH and corticosterone, with the pulse of ACTH occurring in advance of CORT pulse. Increasing the constant dosage of CRH had the effect of pushing the trough levels of CORT toward a ceiling of maximal production, eventually attenuating the rhythmicity. It has been estimated that biosynthesis of CRH requires 60–90 min, thus making it likely that hormonal feedback regulation of CRH production is more involved in recuperation rate or adaptation to chronic stress than initial response (Watts, 2005). Together these findings support the concept of dynamic equilibrium of the HPA axis rather than a controlled steady state, with rapidly fluctuating dynamics functioning to maintain plasticity and responsiveness of the system. It has been proposed that pulsatile dynamics also may serve to filter out random low-level stimuli to avoid unnecessary activation of the system (Rankin et al., 2012). In support of this idea that ultradian dynamics govern the degree of HPA activation, the magnitude of stress response has been shown to depend on whether a stressor occurs during a rising or falling ultradian phase of hormone release (Windle et al., 1998; Sarabdjitsingh et al., 2010).

## Disruptions of GC Signaling Dynamics are Associated with Disease

Timing of gene expression programs is one way in which organisms may maximize the efficient usage of nutrients, cofactors, and other macromolecules, thereby enhancing fitness (Liu et al., 1995; Klevecz et al., 2004; Lloyd and Murray, 2005; Tu et al., 2005). Conversely, disruption of this timing comes at a cost to fitness, and scenarios where GC dynamics become disrupted—either due to chronic stress, interference with circadian cues (e.g., shift work, jet lag), or a disease condition—are associated with multi-systemic disorders, including immune, psychological and metabolic syndromes (Silverman and Sternberg, 2012; Oster et al., 2016).

### Loss of Circadian Rhythmicity

Perhaps the best-known example of attenuated diurnal cortisol levels is in Cushing’s Syndrome, where a pituitary or adrenal tumor leads to cortisol overproduction and loss of rhythmicity. Due to the important role of the GR in most tissues, Cushing’s hypercortisolemia usually presents with multiple comorbidities including metabolic syndromes, hypertension, osteoporosis, cognitive decline, defects in wound healing and increased risk of infections. This collection of maladies is also associated with aging, and it also happens that changes in HPA axis rhythmicity have been associated with aging. The most consistently reported change in CORT levels with age has been an elevation of the evening nadir level in aged individuals, although total increase, circadian phase advancement, and sex dependent differences have also been reported (Van Cauter et al., 1996, 2000; Gust et al., 2000). Interestingly, elevated trough level of CORT can also result from mild chronic stress and cause metabolic imbalance (Dallman et al., 2000). Higher CORT nadir and thus reduced diurnal slope have also been reported in Type 2 diabetics (Hackett et al., 2014).

It is technically challenging to measure basal hormone levels in a stress-responsive system with ultradian rhythmicity. This, along with variations among populations and experimental designs, may be why studies suggest depressive adolescents exhibit a flatter diurnal profile but there is a lack of consensus on how diurnal cortisol is effected in adults with depression (Joseph and Golden, 2017). However, patients with Cushing’s Syndrome are at a high risk (50–80%) for depression, as are patients treated with oral synthetic glucocorticoids widely prescribed for their anti-inflammatory properties (Fardet et al., 2012). Meta-analysis also strongly indicates that elevated cortisol is associated with depression, with the effect being larger in older patients (Stetler and Miller, 2011). This age-dependent finding highlights the potential of interactions to exist among conditions associated with HPA hyperactivity, and the difficulties that can arise in untangling cause and effect.

### Altered Ultradian Pulsatility

With ultradian pulsatility underlying the circadian profile of cortisol, it is not surprising to find that increased ultradian pulse amplitude has also been described in some cases of acute depression (Linkowski et al., 1987; Oster et al., 2016). Adjuvant-induced arthritis has also been shown capable of inducing increased ultradian GC pulse amplitude in rats (Windle et al., 2001). Windle et al. described this effect of mycobacterium-induced inflammation as an increase in frequency of pulses (in terms of more corticosterone pulses counted *per* 24 h). However, this increase in “frequency” was driven mainly by the occurrence of measurable pulses of CORT throughout the day in treated animals, whereas in controls CORT pulses were not detectable during the circadian trough period. When detected, pulses occurred with an ultradian period of ~33 min regardless of treatment; however, amplitude remained high throughout the day in arthritic rats whereas pulse amplitude had a circadian profile in controls. In humans, ultradian cortisol pulse frequency has been shown to remain relatively constant even at low hormone levels (Young et al., 2001). That pulsatile CORT release is detectable even during circadian nadirs and may be induced to maintain a constant high amplitude by chronic stimuli suggest that the HPA axis is a system that oscillates even during circadian troughs rather than entering a static inactive state.

It has been suggested that oscillating biological systems may be produced by evolutionary tuning of discreet pulsatile responses which are well-suited for adapting to *changes* in environment rather than to the level of an environmental input itself (Cheong and Levchenko, 2010). Negative feedback regulation can be used to enhance responsiveness to stimulus but can also generate overshoot and unstable oscillations. Additional feedback and feedforward regulatory elements can be combined to stabilize and tune such a system to achieve an optimal combination of responsivity and stability (Ma et al., 2009; Reeves, 2019). The HPA axis may thus be thought of as an oscillating system primed to generate adaptive, pulsed responses. These dynamic characteristics can confer benefits such as enhanced responsivity and adaptation, amenability to entrainment and/or filtering out of noisy/trivial activating stimuli (Reeves, 2019). However, as noted above, the HPA axis appears also to have some inherent vulnerability to repeated or chronic activation.

## The Allostatic Model of Pathogenesis

**Figure 4:**
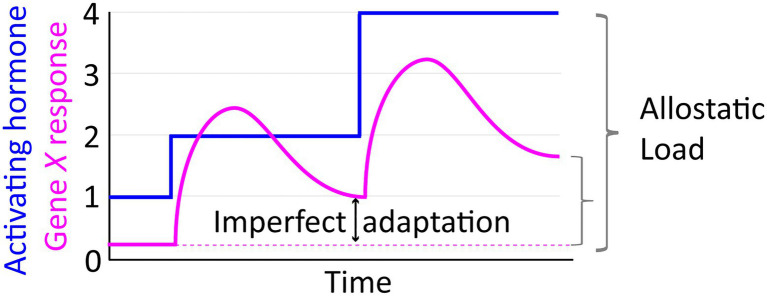
The allostatic load concept illustrated in relation to an idealized depiction of transcriptional response dynamics manifesting imperfect adaptation to stepwise increases in activating hormone.

A useful concept for relating GC and HPA dynamics to disease states is that of ‘allostasis,’ i.e., stability through adaptation (McEwen, 1998; Picard et al., 2014). Building from the concept of homeostasis and physiological set points optimized for fitness, allostasis emphasizes the work required by a system (such as the HPA axis) to maintain those parameters. In response to many types of stress, biological systems have a capacity to mount transient adaptive responses. As an idealized generic example, consider stress-induced stepwise increases in hormone level that activate transcription of a target Gene *X* (Figure 4). From a systems design perspective, this response would be considered perfectly adaptive if transcription of *X* increases transiently and then returns to the pre-stimulus level (Shi et al., 2017; Reeves, 2019). The pulsed response of *X* might also resolve with a new steady state of transcriptional output (e.g., higher than the pre-stress level), termed “imperfect adaptation” (Figure 4). The allostatic model of disease posits that if adaptive responses are repeatedly or chronically activated, they may culminate in an imbalanced systemic state characterized by a new homeostatic set point (i.e., set-point drift), referred to as an “allostatic load” that is unhealthy (Figure 4). The regulation of blood sugar by GCs is an illustrative example. During the acute stress response, GCs cause release of glucose by the liver while simultaneously antagonizing insulin signaling and decreasing glucose uptake in skeletal muscle and adipose tissue (Kuo et al., 2015). The net result of this is increasing glucose availability to the brain, which relies almost exclusively on glucose for its high energy demands (~20% of glucose metabolized by an organ which accounts for ~2% of body mass; Mergenthaler et al., 2013). Chronic stress, however, can contribute to allostatic load *via* chronic hyperglycemia that leads to increased inflammation, insulin resistance, and eventual development of diabetes and related metabolic syndromes.

### The Allostatic Load Index

The progression of disease due to allostatic load can be divided into three phases. First, a change in a primary mediator of allostasis (such as cortisol) that results in a secondary effect on physiology (such as hyperglycemia) followed by a tertiary manifestation of a clinically recognized disorder (such as diabetes). To translate the concept of allostatic load into a clinical/biomedical tool, various permutations of an Allostatic Load Index have been tested using combinations of measurable primary and secondary biomarkers to predict clinical outcomes. Since cortisol is a primary mediator of adaptation to stress, cortisol measurement was included among 10 measurements in the original Allostatic Load Index, developed as part of a MacArthur study on successful aging (Seeman, 1997). In this study of a selected ‘high-functioning’ elderly population, scoring poorly on the Allostatic Load Index was associated with (and predictive of) poor physical health and cognitive decline, with cortisol level contributing to the index’s predictive capacity (Karlamangla et al., 2002). Encouragingly, decreases in allostatic load were found to reduce risk of death in a follow-up study (Karlamangla et al., 2006). Since the initial MacArthur study, cortisol has remained frequently (but not always) included in subsequent studies using variations of the index, alongside other neuroendocrine, metabolic, and immune markers and physiological measurements (blood pressure and waist-to-hip ratio, for example; Juster et al., 2010).

### Allostatic Load in Working Populations

As work is considered a common source of stress, more than a dozen studies have looked at Allostatic Load in working populations, and high Allostatic Load has been shown to correlate with reported stress level, effort-reward imbalance, and low safety and socio-economic standing (Mauss et al., 2015). However, considerable variations in design and lack of a standardized index among these studies—dozens of different biomarkers being used in at least one study—limits meta-analysis. In one such study, low cortisol was associated with symptoms of burnout but not depression (Juster et al., 2011), but broadly speaking variations in populations, study designs, timing and frequency of cortisol collections and sample type (cortisol can be measured in urine, saliva, plasma, or hair) have led to inconsistent results and lack of consensus on the specific contribution of cortisol to Allostatic Load in working populations (Dowd et al., 2009). Nevertheless, it is worth considering that work-related stress may contribute to differential cortisol levels as well as other markers/outcomes of allostatic load.

### Early Life Allostatic Load and Long-Term Consequences

A number of studies have linked early-life stress (e.g., traumatic childhood experiences or development in adverse environments) with measurably increased Allostatic Load Index in children (Katz et al., 2012), and adverse childhood events have been shown to cumulatively increase long-term risk of mental, physical, and behavioral disorders (Anda et al., 2006). Quality of care has been shown to have an effect on the diurnal cortisol profile in pre-school aged children, who display the expected diurnal decrease in cortisol when at home but often experience an increase in cortisol from morning to afternoon when at daycare, particularly if care quality is lower (Tout et al., 1998; Dettling et al., 1999; Watamura et al., 2009). In one study, this elevated afternoon cortisol in school children was found to correlate with lower antibody production (Watamura et al., 2010). School-age children from lower socio-economic backgrounds have been shown to have elevated cortisol levels compared with children from more affluent families (Lupien et al., 2000). Enriched environments have been shown in some instances to decrease cortisol, while the emotional stress of having depressed parents or being rejected by peers leads to cortisol elevation (Gunnar and Donzella, 2002).

Although the long-term effects of elevated cortisol during childhood are not well understood, exposure to early-life stress has been shown to modulate the activity and responsivity of the HPA axis later in life. But as with the effects of allostatic load in adult populations, the outcomes appear context-dependent (Van Bodegom et al., 2017). For example, while mild-to-moderate early life stress can lead to HPA axis hyperactivity as seen in depression, more severe trauma can lead to hypo-responsiveness, with developmental timing of stress exposure also factoring into outcomes. Nevertheless, low socio-economic standing in childhood has been linked to an increase in basal cortisol in adulthood, independent of adult socio-economic standing and other lifestyle factors (Miller et al., 2009). This was accompanied by a decrease in GR-target gene expression in immune cells and an increase in markers of inflammation, supportive of the authors’ hypothesis that early-life stress promotes a “defensive” phenotype that could be beneficial during acute stress but more susceptible to chronic disease. Low childhood socioeconomic standing has also been linked to increased infection susceptibility, risk for cardiovascular disease, and overall mortality (Cohen et al., 2004; Galobardes, 2004; Galobardes et al., 2006). Thus, stress experienced during childhood is linked to poor long-term health outcomes as well as altered cortisol production during childhood and into adulthood, strongly indicating that early-life programming of the HPA axis contributes to disease risk.

## Pre- and Peri-Natal Stress, Glucocorticoid Exposure, and Developmental Programming of Stress Responsivity

Given the evidence that elevated stress levels in childhood can have long-term effects on HPA function and overall health, it may come as no surprise that abundant research indicates that mal-adaptive programming of the HPA axis can begin *in utero*. Due to the rapid growth and development occurring during the earliest stages of life, the pre- and peri-natal periods are generally considered to be times of high plasticity and vulnerability to intervention.

Endogenous GCs are crucial for early mammalian development, aiding the maturation of fetal organs. In 1969, it was reported that synthetic GC-induced inflation of lungs in premature lambs (Liggins, 1969). Mice lacking a GR die soon after birth due primarily to defects in lung surfactant production and respiratory distress (Cole et al., 1995). In humans, respiratory distress is a leading cause of death in pre-term newborns, and so synthetic GCs are regularly administered to hasten lung maturation when there is risk of a pre-term birth (Murphy et al., 2006). As will be discussed in the following sub-sections, development of the brain and the HPA axis itself are also finely regulated by GCs and elevated exposure can be detrimental.

### Prenatal Exposure to Synthetic Glucocorticoids

The use of synthetic GCs has been endorsed by the NIH to aid fetal maturation in pregnancies at risk for premature delivery (Gilstrap et al., 1995; Gilstrap, 2001; Harris and Seckl, 2011). Though there are definite benefits of administered GCs for survival of premature infants, for pregnancies carried to term there may be negative effects, and the use of multiple courses is also controversial (Roberts et al., 2017; Räikkönen et al., 2020). How prenatal administration of synthetic GCs affects long-term health is not yet fully understood, as the practice only began in earnest in the 1990s. There are however multiple reports of adverse associations. Antenatal treatment with synthetic GCs has been reported to cause higher blood pressure in adolescents and elevated insulin in 30-year-old adults (Doyle et al., 2000; Dalziel et al., 2005). Decreased head size, hyperactivity, and increased distractibility were reported in children treated with repeated antenatal courses of betamethasone (French et al., 2004). Pre-mature infants treated for respiratory distress with DEX after birth have been reported to have less cerebral grey matter at term, and to have delayed growth, impaired cognitive, neuromotor function and IQ, and increased risk for disability at age 8 (Murphy et al., 2001; Yeh et al., 2004). In one study, multiple courses of synthetic GCs were associated with increased risk of neurosensory disability at 5 years of age, relative to risk from a single course treatment (Asztalos et al., 2013). Another study that found multiple courses of antenatal GCs to decrease newborn respiratory distress reported no increased risk of neurosensory disability in follow-ups, but did find increased risk for hyperactivity at age 2 and borderline associations with behavioral regulation and ADHD in 6–8 year olds (Crowther et al., 2006, 2007, 2016).

In rhesus monkeys, dose-dependent degeneration of neurons was reported after antenatal DEX (Uno et al., 1990). In sheep, repeated maternal doses of betamethasone increased lung function in pre-term lambs, but also led to lower birth-weight, a significant indicator of long-term health risk in human babies (Ikegami et al., 1997; Newnham et al., 1999). Contributing to the decrease in ovine birthweight due to prenatal GC exposure was the significant decrease in weight of a number of organs including the thymus, kidney, liver, and brain [where decreased myelination was reported as well (Huang et al., 2001)]. Interestingly, lambs given one betamethasone dose during the third-trimester were found to have increased cortisol production in response to CRH at 1 year of age, while those receiving the initial dose followed by three additional antenatal doses did not (Sloboda et al., 2002). In a rat cell culture model of neuronal development, a subclinical dose of DEX was found to enhance cell viability at the expense of DNA synthesis and neuronal differentiation, as well as promoting adrenergic over cholinergic fate (Jameson et al., 2006). Another cell culture model using human hippocampal progenitor cells found that chronic DEX treatment during proliferation/differentiation led to long-lasting methylation changes in poised bi-valent promoters and an increased transcriptional response to subsequent acute challenge (Provençal et al., 2020). Intriguingly, the largest long-lasting effect (−20.1% methylation compared to control after 20-day) was found in the locus of the GR chaperone *FKBP5*, a key mediator of stress-induced developmental programming for which variants have been identified with GRE sequence polymorphisms that confer differential susceptibility to the long-term effects of early life stress (Binder et al., 2004; Klengel et al., 2012). In adult male mice, PVN-specific forced overexpression of *Fkbp5* has recently been shown to induce a “stress-like phenotype” including elevated basal CORT (Häusl et al., 2021).

### Fetal Exposure to Maternal Cortisol

While synthetic GCs administered to the mother are believed to freely cross the placental barrier, the exposure of fetal tissues to endogenous CORT is limited by the expression of the enzyme 11ß-hydroxysteroid dehydrogenase type 2 (HSD2), which converts endogenous GC molecules to inactive ketone forms. This enzyme is expressed dynamically in fetal tissues throughout development as well as in the placenta where it inactivates the majority of maternal CORT (Brown et al., 1996; Shams et al., 1998). However, maternal and fetal CORT levels are correlated, and even a small percentage of maternal cortisol passing through the placental barrier can double the normal fetal blood concentration, which is much lower than that of the mother (Takahashi et al., 1998; Zijlmans et al., 2015). Chronic maternal stress may also decrease the efficacy of the CORT barrier, as in animal models chronic stress has been shown to decrease HSD2 expression and increase fetal exposure to maternal CORT (Mairesse et al., 2007; Jensen Peña et al., 2012). The placenta also produces CRH when stimulated by maternal cortisol (and by fetal cortisol once the fetal HPA axis develops). This placental CRH leads to fetal ACTH and CORT production, in addition to that produced by the mother. Importantly, in contrast to the negative feedback between CORT and hypothalamic CRH production, CORT has a positive feed-forward effect on placental CRH which increases steadily throughout pregnancy and rapidly as birth nears, and may play a role in timing parturition (Majzoub and Karalis, 1999).

### Long-Term Health Effects of Pre- and Peri-Natal Stress

It is estimated that ~15% of pregnant women experience extended psychiatric conditions such as depression and anxiety (Fisher et al., 2012). Although no broad consensus exists for how these complex emotional states relate to cortisol levels, risk factors include low socio-economic standing and lack of supportive relationships, highlighting a similar etiology with chronic psychosocial stress and allostatic load. Thus, the prevalence of these states may offer a reasonable (and likely conservative) proxy for prevalence of chronic maternal stress. As noted above, maternal stress mediators including cortisol can be transferred to the developing fetus, and there is evidence that a maternal state of chronic stress programs long-term changes in function of the offspring’s HPA axis and disease risk (Harris and Seckl, 2011; Van Bodegom et al., 2017; Van Den Bergh et al., 2017). One theory is that maternal GCs serve to tune the fetal HPA axis to respond appropriately to the environment outside the womb, but that this can be mal-adaptive if the environment later in life is incongruent with the developmental one. This has been called the “Match/Mismatch” theory, and is essentially an evolution of the “Thrifty Phenotype” or Barker Hypothesis regarding the Developmental Origins of Health and Disease, although Barker focused primarily on negative long-term effects of early-life malnutrition (Hales and Barker, 2001; Gluckman et al., 2007).

#### Human Studies

The Barker and Match/Mismatch theories place fetal programming at the origin of long-term health and relate to the Allostatic Load model of disease, as biomarkers of Allostatic Load in a mother (e.g., cortisol) clearly influence fetal development. Maternal salivary cortisol level during a stressful prenatal procedure (amniocentesis) has been shown to be predictive of birthweight (Ghaemmaghami et al., 2014). As birthweight is a strong indicator of long-term health, this indicates that reactivity of the maternal HPA axis may predict future offspring health. This also demonstrates the overlapping domains of stress and metabolic programming. More direct evidence of an HPA axis programming effect exists. In one study, high amniotic cortisol at mid-gestation was correlated with a higher basal cortisol level at 17 months of age as well as an inverted cortisol response to separation from the mother (O’Connor et al., 2013). Correlation between mid-gestational maternal and neonatal ACTH and cortisol levels was demonstrated in another study, although a prevalence of major depressive and bipolar disorders in that study population may limit generalization (Smith et al., 2011). In a more broadly inclusive study of mother/infant dyads, elevated cortisol response and blunted recovery to heel prick procedure were found in infants of mothers with higher cortisol levels measured at mid-gestation (Davis et al., 2011). Higher maternal perception of stress during early/mid pregnancy also correlated with more aroused infant behavior during recovery from the heel prick. Pregnancy-specific prenatal anxiety (e.g., fear that the child will be born with disability) has also been linked to infant cortisol reactivity (Tollenaar et al., 2011).

The effects of maternal/prenatal stress on offspring HPA axis function may extend through childhood, as modest correlations with a child’s cortisol level on the first day of school and adolescent diurnal cortisol levels have also been measured (Gutteling et al., 2005; O’Donnell et al., 2013). Neurobehavioral phenotypes in childhood have also been linked to maternal/prenatal stress and cortisol levels. These include ADHD (as with synthetic GC administration) and autistic traits (Ronald et al., 2011) as well as anxiety (Davis and Sandman, 2012). One retrospective study using plasma samples frozen between 1959 and 1966 has reported an association of higher maternal cortisol during the third trimester with decreased offspring IQ at age 7 (Lewinn et al., 2009). However, the reliance of this study on dated questionnaires, and the lack of controls for important factors such as sampling time of day and food intake (participants had not fasted) calls for caution in interpreting these results. Decreased grey matter at 6–9 years of age has also been linked to mid-gestation maternal anxiety levels (Buss et al., 2010), but another recent study has shown that prenatal exposure to higher cortisol (while within the normal range) during the third trimester leads to thicker brain cortex and improved performance on intelligence tests of children in the same age group (Davis et al., 2017). This fits with the fetal maturation role of rising GCs in late pregnancy. Yet another study found that multiple major stress events during pregnancy led to lower reading scores in 10-year-old female offspring but higher reading and math scores in male offspring (Li et al., 2013). It is intriguing to note that maternal cortisol level during early (but not late) pregnancy has been linked to a significant increase in amygdala volume in 7-year-old female offspring but a trend toward increase in hippocampal volume in male offspring of the same age, effects with potentially opposite effects on HPA axis regulation (Buss et al., 2012). Similarly, sex-specific differences were found in assessments of executive function in 6-to-9 year old children, where level of Pregnancy-Specific Anxiety during the first two trimesters (but not third) accounted for a decrease in inhibitory control in girls but not boys (Buss et al., 2011). Studies indicate that Pregnancy-Specific Anxiety occurs most often early on and decreases over the course of gestation, while at the same time risk for depression increases (Sandman et al., 2016), and these data highlight the potential for complex and dynamic interactions of sex, timing, and nature of maternal stress during early development.

Although many of the above-noted correlations between maternal stress/cortisol and offspring outcomes could be attributable to prenatal exposure to maternal GCs, they could also be driven by underlying genetics, inherited predisposition, or learned/acquired behavior. A few human studies have attempted to separate the contributions of prenatal environmental stress from genetics. A study of *in-vitro* fertilized offspring found significant effects of maternal stress level on birth weight (lower) and antisocial behavior (higher) in both related and cross-fostered mother–child pairs. Conversely, an effect of prenatal stress on ADHD was only found in related pairs, indicating a genetic driver, while the effect on child anxiety level was found to be mediated by the mother’s post-natal mood (Rice et al., 2010). In another study, child cognitive development was found to suffer a deficit in mothers who had increased objective exposure to life-stress related to a severe ice storm during their first or second trimester (King and Laplante, 2005). A Finnish study using maternal fear of exposure to radiation from the Chernobyl disaster as the source of prenatal stress found that individuals in the second trimester of fetal development at the time of the disaster were at a greater risk for depression and ADHD as adolescents (Huizink et al., 2007). However, the underlying population here was selectively enriched for those at higher risk of familial alcoholism and so may not be broadly representative. There were also potentially confounding differences in socioeconomic status and weight at birth among study populations. Although the authors pointed to higher birth weight in the prenatally stressed group as evidence of good health and no ill effects of actual radiation exposure, this may not necessarily be the case. A different study found that prenatal stress exposure due to maternal bereavement increased the risk of overweight/obesity in adolescents (Li et al., 2010). In all, human studies point convincingly to effects of prenatal stress on long-term health, however they also illustrate the difficulties of performing human studies with enough experimental control to pinpoint cause and effect. Further, none of these mentioned studies which attempted to untangle developmental programming from hereditary factors measured cortisol or affiliated biomarkers directly. Lastly, at this time few if any studies have looked at how/whether the effects of perinatal HPA axis programming extend into late adulthood.

#### Studies in Mammalian Animal Models

Studies using animal models have begun to elucidate mechanisms of fetal HPA axis programming by maternal stress. Chronic restraint stress in pregnant rats has been shown to decrease placental HSD2 expression, thus increasing fetal exposure to maternal GCs (Mairesse et al., 2007; Jensen Peña et al., 2012). Fetuses subjected to the prenatal restraint stress regime also had lower body weight, less pancreatic beta-cell mass, and lower ACTH and blood glucose. Higher methylation of CpG dinucleotides in the *Hsd2* promoter in stressed placentas was shown to coincide with the decrease in placental HSD2 expression. This was also correlated with lower *Hsd2* promoter methylation in the fetal hypothalamus, evidence of epigenetic programming. Chemical inhibition of HSD2 in pregnant rats also caused decreased birthweights, as well as increases in adult offspring blood glucose and insulin. Importantly, these effects were dependent on the mother having intact adrenal glands (Lindsay et al., 1996).

There is abundant evidence that developmental calibration of the HPA axis continues in the period directly following birth, during which mammalian young experience a period of stress hypo-responsiveness that may protect brain development from the detrimental effects of high GCs. Though human infants are born with an intact cortisol response to stressor (e.g., separation from mother for doctor’s examination), this responsiveness decreases between 2 and 6 months of age and remains low until ~15–18 months. But this state of suppressed cortisol is dependent on parental caregiving and attentiveness (Gunnar et al., 1996a,b; Gunnar and Cheatham, 2003). In the rat, the stress hypo-responsive period occurs from roughly postnatal days 3–14 and is maintained by maternal care (i.e., licking, grooming, and nursing behavior) and disrupted by prolonged maternal separation, which increases pup HPA activity (Rosenfeld et al., 1992). Further, rat dams can be segregated by the level of care they show newborn pups under undisturbed circumstances into “high-licking” and “low-licking” subgroups. Adult offspring of “low-licking” dams (i.e., those receiving less maternal attention) show HPA hyperactivity in terms of both ACTH and GC levels, increased *Crh* mRNA, and decreased GR expression in the hippocampus likely indicating impaired negative feedback regulation of the HPA axis (Liu et al., 1997). This decreased expression of hippocampal GR was subsequently shown to be accompanied by increased CpG methylation in the *Nr3c1* promoter, suggesting epigenetic programming of the HPA axis by maternal care (Weaver et al., 2004). Intriguingly, both hippocampal GR expression and HPA hyperactivity could be rescued in the adult offspring by cerebral infusion with an inhibitor of the histone deacetylation complex.

In rat cross-fostering studies, maternal behavior and level of fearfulness were shown to be transferred to rat offspring through maternal care (Francis et al., 1999). Behavioral defects were accompanied by decreased levels of hippocampal GR and increased CRH. All of these outcomes, however, could be reversed by daily mild handling of pups of low-licking mothers, a protocol that has been long recognized to impart a level of stress resilience to pups (Levine and Lewis, 1959). Handling also rescued the reduced expression of anxiety-inhibiting benzodiazepine receptors in the amygdala of offspring of low-licking dams. In a study where *pre*-natal stress (repeated maternal restraint with bright illumination) was found to induce prolonged GC production in adults in response to stress, as well as increased anxiety behavior, mild postnatal handling was found to have the opposite effect on behavior (Vallée et al., 1997). These data suggest that early-life stress exposure is hormetic [with an inverted U-shaped response similar to direct GC exposures (Lupien et al., 2005; Pratsinis et al., 2006; Monaghan and Haussmann, 2015; Gopi and Rattan, 2019)], conferring resilience or other benefits below a threshold, but harmful if too severe.

The susceptibility to effects of pre- and peri-natal stress shows dependence on genetics. In mice for instance, C57BL/6J dams have been reported to display a measurably higher level of baseline maternal care than BALB/c, and this correlates with decreased anxious behavior and cortisol response to stress in their offspring (Priebe et al., 2005). Cross-fostering of BALB/c pups to B6 dams significantly decreased anxiety-like behavior in adult offspring, whereas cross-fostering in the opposite direction produced elevated basal CORT in B6 mice fostered by BALB/c dams but not a significant increase in anxious behavior, likely indicating some inherent resilience (Priebe et al., 2005). Strain-specific effects of pre-natal stress on CORT and hippocampal gene expression response to stress have also been reported in rats (Neeley et al., 2011). In human studies, prenatal stress has been shown to interact with gene variants involved in GC and neuronal signaling pathways to modulate risk of psychiatric and behavioral disorders (Abbott et al., 2018).

#### Studies in Zebrafish

Over the previous two decades, the zebrafish, *Danio rerio*, has emerged as a powerful model organism for studying GC signaling, with most such studies having taken place in just the last 5 years. Several intrinsic characteristics make zebrafish a favored organism among developmental biologists. These include the large number of offspring produced (up to 200–300 per mating pair), external fertilization and embryonic development, and optical transparency of the embryo. Zebrafish embryos also develop rapidly, going from single cell to free-swimming larva in a few days. Finally, the large one-cell stage of the embryo provides a relatively easy target for micro-injection protocols, facilitating genetic manipulation techniques such as gene knockdown using morpholino oligonucleotides, transposon-mediated transgenics, and CRISPR-mediated gene editing.

Despite evolutionary distance, 70% of zebrafish genes having identifiable human orthologs and vice versa (Howe et al., 2013). Due to multiple whole genome duplications in vertebrate ancestry, including one duplication since the divergence of teleost and tetrapod lineages, many human genes have two or more zebrafish orthologs. Approximately 2,900 zebrafish genes have such duplicates, but in contrast to many teleosts that have been studied, zebrafish have retained only one copy of the *nr3c1* gene that codes for the GR, the DNA- and ligand- binding regions of which are highly conserved across vertebrata (Alsop and Vijayan, 2008a). As in humans, alternative splicing results in a lowly expressed beta isoform of the GR protein in zebrafish; however, the function of this minor isoform remains unresolved in both species (Chatzopoulou et al., 2017). Also, in common with its human ortholog, the zebrafish *nr3c1* gene contains multiple potential translational start codons, and there is some evidence that these are functional (Gans et al., 2020). Upstream in the GC signaling pathway, zebrafish have only one copy of the *crh* gene, like mammals and unlike many fish. Zebrafish do retain a duplicate copy of the *pomc* gene that codes for the proopiomelanocortin protein that is cleaved to produce ACTH. However, the cleavage site of one paralog has been mutated so that only one gene generates functional ACTH (Alsop and Vijayan, 2009). It is a bit of a curiosity that zebrafish have lost duplicate copies of genes involved in each level of the HPA axis, however, it makes *D*. *rerio* a tractable model for studying the vertebrate neuroendocrine stress system.

The tissues of the HPA axis and their functions are essentially conserved from humans to zebrafish, with the caveat that in *D. rerio* and other teleosts, cortisol is produced by interrenal cells dispersed throughout the head kidney rather than by a gland proper. Hence, the HPA axis is referred to as HPI (Interrenal) axis in fish (Figure 1). However, more notable differences between zebrafish and mammalian corticosteroid signaling do exist. Teleosts lack the *cyp11b2* gene and thus do not produce the mineralocorticoid aldosterone (Bridgham et al., 2006). The lack of this competing ligand may make the MR relatively more responsive to cortisol levels in zebrafish (although the MR has affinity for other steroids as well) and work comparing GR and MR mutant zebrafish has shown that the two receptors work together to regulate the response to acute stress (Faught and Vijayan, 2018; Lee et al., 2019). Cortisol transport through the blood also may be significantly different in zebrafish than mammals. In mammals, albumin accounts for a large percentage of plasma protein and, along with the dedicated corticosteroid binding globulin (CBG), binds roughly 90% of CORT creating a reservoir of inactive hormone in the plasma. Although the majority of protein types detected in zebrafish plasma are also found in human plasma, zebrafish lack albumin and CBG (Li et al., 2016). Zebrafish do have a Vitamin D binding protein (*dbp*) with some homology to albumin (Noël et al., 2010), but whether this or other zebrafish blood proteins play a role in transporting cortisol is unknown.

Several zebrafish mutants lacking functional GR have been produced (reviewed in Dinarello et al., 2020), which have the advantage of being viable throughout adulthood, in contrast to mammalian systems wherein loss of the receptor results in neonatal death through mechanisms previously described. Unsurprisingly however, lower survival has been noted in mutants at zygotic/embryonic (Gans et al., 2020; Maradonna et al., 2020) and post-larval stages (Facchinello et al., 2017). In 2012, a GR mutant, *s357*, was identified in a behavioral screen for depressive-like behavior (Ziv et al., 2013). A single nucleotide polymorphism was found to eliminate DNA-binding (DBD) activity of the GR and abolish transcriptional regulation of target genes (though preserving the ability of the GR to bind cortisol and translocate into the nucleus, as well as potential for protein–protein interactions). These mutants displayed HPI hyperactivity, with elevated basal and stressed levels of CORT and basal *crh* and *pomc* expression, as well as lack of suppression of CORT after treatment with DEX (i.e., the DEX suppression test) indicating that negative feedback regulation of the HPI axis was not intact. Behavioral rescue in mutants with anti-depressant and anxiolytic drugs revealed a crosstalk between genomic GR signaling and GABA- and serotonergic circuitry, but not norepinephrine or dopamine pathways.

GR *s357* mutants have been found to suffer tissue defects in heart (reduced trabecular network) and intestine (shortened villi) that progress with age, and increased sub-cutaneous fat stores (Facchinello et al., 2017). Lower heart rate and blunted inflammatory gene response were also measured in mutant larvae. This blunted inflammatory response is particularly intriguing, as we have found similar defects in inflammatory gene expression in adult WT fish exposed to elevated cortisol only during early development (Hartig et al., 2016). The latter inflammatory defects were measured in response to both tail-fin injury and response to lipopolysaccharide (LPS, mimicking bacterial infection). Subsequently, we found broad similarities in the transcriptomes of vehicle-treated GR^−/−^ larvae and WT larvae treated with chronic CORT, suggesting that chronic cortisol treatment induces resistance to genomic GC signaling (Gans et al., 2020). Mutation of GR and treatment with exogenous GCs have also been shown to alter zebrafish leukocyte migration in response to injury, although the reported effects differ depending on timing and nature of treatment and injury (Chatzopoulou et al., 2016).

With the advent of CRISPR, several laboratories have generated GR mutant zebrafish lines to further exploit the usefulness of zebrafish to investigate vertebrate GC signaling (Dinarello et al., 2020). Like the *s357* strain, mutants with a 7-bp deletion in exon 2 leading to a truncated protein display hyper-cortisolemia. These mutants were also found to have increased muscle mass and protein synthesis, along with decreased protein catabolism and abrogated blood glucose response to stress (Faught and Vijayan, 2019a). These findings beautifully illustrated the role of the GR in systemic energy balance, and that restriction of glucose uptake by muscle cells is key to elevating blood glucose in response to challenge, likely as fuel for the brain. The same mutant line was also used alongside a MR knockout to demonstrate the coordination of the two corticosteroid receptors in maintaining postnatal growth, insulin and triglyceride levels (Faught and Vijayan, 2019b, 2020).

Like humans, zebrafish are diurnal, and display circadian behavior patterns that are entrained by rhythmic stimuli such as light cycles or feeding schedules. In this context, it has been shown that treatment with a high level of the synthetic GC prednisolone increases the amplitude of expression of a luciferase reporter for the circadian *per3* gene, and that this increase of expression is dependent on a GR with functional DNA binding. We have similarly found that chronic treatment with cortisol induces a modest increase in expression of the paralogous *per1a* (Gans et al., 2020). Elsewhere, a functional GR has been shown necessary to entrain circadian feeding behavior in zebrafish (Morbiato et al., 2019), to be necessary for retinal light adaptation (Muto et al., 2013). Somewhat reciprocally, eyeless mutants for the retinal homeobox gene *rx3* are cortisol deficient (Weger et al., 2016). Comparison of transcriptomes of these *rx3* mutants with WT larvae indicated altered expression of roughly half of rhythmically expressed genes (nearly a quarter of all genes measured). These affected genes were enriched for involvement in metabolism and cell cycle regulation. Strikingly, the rhythmic expression of more than half of the affected genes was rescued by treatment with a constant concentration of DEX, indicating that rhythmicity of GC levels may be more important for some downstream processes than others.

Given the high level of conservation in the GC signaling axis and the ease of manipulating externally developing embryos, several laboratories have begun to use zebrafish as a model to examine the developmental programming effects of elevated cortisol. This work has built on work done around the turn of the 21st century that established the basic timing of HPI axis development in *D. rerio*. Maternal GR (*nr3c1*) mRNA and cortisol are deposited in the embryo but are largely depleted by the time of hatching, (~48 h post-fertilization, hpf), at which point embryonic transcription is well underway and tissues of the HPI axis are established and functional (Alsop and Vijayan, 2008a,b). We have found that the expression of GR targets *fkbp5* and *klf9* parallels that of *nr3c1*, being relatively high immediately after fertilization, then decreasing to a minimal level on days 2–3 post-fertilization before rebounding again by day 4 (Gans et al., 2021). Though embryos can produce a basal level of cortisol by 48 hpf, a measurable cortisol response to stress is not mounted until roughly 4 days post-fertilization (4 dpf). The lapse between cortisol productivity and stress responsiveness appears to be due to lack of neurodevelopment and thus sensitivity to stimuli, as CRH and ACTH are also produced by 1–2 dpf and are thus not limiting factors. This stress hypo-responsive period is reminiscent of that found in mammals. Also reminiscent of mammalian development, maternally deposited GR may play a role in inflation of the zebrafish swim bladder (homologous to the mammalian lung) as defects in inflation were found alongside other developmental defects following blocking translation of maternally deposited GR transcript with morpholino (Pikulkaew et al., 2011).

There is some limited evidence that elevated maternal cortisol in zebrafish results in more cortisol deposited in the embryo, and as in mammals there appears to be protection in place to limit this occurrence. Five days of fasting stress were shown to increase whole body cortisol ~4-fold in female adults (Best, 2015). Embryo cortisol content however was only significantly increased in embryos laid by stressed females toward the end of a 10-day breeding period following the fast. In another experiment, female fish were fed a diet spiked with cortisol for 5 days and then spawned over the course of the following 10 days. Cortisol-fed females produced embryos with significantly higher cortisol levels only on the third day after the end of their feeding regime, but not the days before or after (Faught et al., 2016). One possibility suggested by the authors was that cortisol incorporation into the eggs would be maximal only in oocytes undergoing yolk body accumulation during the treatment window. Given the range and variability of embryonic cortisol measured, alternate explanations could include genetic variability among the females as well as unaccounted for confounding stressors. Repeated spawning—although limited in these studies to every other day for a given fish—is considered by some an unhealthy stress for fish. Unlike fasting stress, dietary cortisol did not result in elevated whole-body cortisol and was cleared quickly. Also, unlike fasting, dietary cortisol did cause significantly higher cortisol in ovaries, potentially indicating different routes of transport/clearance. Subsequently, dissected ovaries treated *ex vivo* with cortisol showed a marked upregulation of *hsd11b2* expression, indicating a protective maternal barrier like that found in mammalian placenta. In a separate study, no effect of dominant/subordinate hierarchical status was found either on maternal whole-body or embryo-deposited cortisol, although offspring of subordinate females produced elevated cortisol and *crh* levels at 48 hpf (Jeffrey and Gilmour, 2016). It should be emphasized here that whole body cortisol measurements represent something of a “black box” in relation to tissue specific rates of cortisol production, utilization, and clearance. We have found evidence that chronic cortisol exposure in early development has long-term programming effects on both overall levels of cortisol and regulation of its tissue transport/uptake in the adult (Hartig et al., 2020).

Given the known ability of maternal cortisol to cross the placental barrier and affect development in mammals, it follows that researchers have begun taking advantage of the external fertilization and development of zebrafish embryos to circumvent some of the complex interactions inherent in mammalian maternal stress models. In zebrafish, direct manipulations of embryonic GC levels can be made easily. While this approach does not model the numerous signaling pathways involved in maternal stress, it does allow for precise control of embryonic exposure to cortisol, and thus addressing the specific question of how that exposure contributes to developmental programming. One method that has been used with some success by the Vijayan Laboratory is microinjection of embryos. Cortisol (32 picograms) delivered in this manner at the one-cell stage was found to lead to an increased rate of heart deformities and under-expression of cardiac genes in the period leading up to hatching, as well as reduced heart-rate in response to a stressor in post-hatch (72 hpf) larvae that displayed mild or no apparent heart deformities (Nesan and Vijayan, 2012). This single injection of cortisol was sufficient to increase the cortisol level throughout the window of the study. For comparison, injection of antibodies to cortisol was used to decrease the hormone’s availability during development (Nesan and Vijayan, 2016). This led to increased *crh, pomc, hsd11b2, star* (Steroidogenic Acute Regulatory Protein), and *nr3c2* (MR) transcript levels at 48 hpf, as well as an accentuated cortisol stress response at 72 hpf, effects all opposite of those seen in embryos injected with cortisol. These results strongly support the theory that cortisol levels during early development programs the HPI axis. Injection with cortisol antibodies also led to an upregulation of *nr3c1* (GR) mRNA and mesodermal defects. A subsequent study by the same group found that injection of 75 pg of cortisol into one-cell embryos led to increased neurogenesis in certain brain regions (including the pre-optic region of the hypothalamus) as well as decreased thigmotactic behavior in 4 dpf larvae (Best et al., 2017). The authors interpreted the reduced thigmotaxis as evidence of increased boldness (or reduced fearfulness). In our experience, larval swimming behavior at 4 dpf is largely reactive to stimulus (and less free-swimming than at 5 dpf when feeding begins), and another interpretation of the data is that the injected cortisol increased sensory input or sensitivity. This idea is supported by the elevated swimming response to light that the authors reported as well as the enhanced neurogenesis in the pre-optic area, a region which receives sensory input.

A study by Higuchi has recently reported that 4 days of maternal fasting led to increased (though not statistically significant) maternal cortisol measured in 10 hpf embryos, and suppressed neurogenesis in the forebrain of larvae as measured at 5 dpf (Higuchi, 2020). No morphological or volumetric differences were found in brains due to treatment. Given that there was a significant increase in the mitotic marker phosphorylated histone H3 reported at 3 dpf, one possibility is that elevated cortisol in fact accelerated the course of neurogenesis. This would agree with, rather than contradict, the report from the Vijayan Laboratory of increased neurogenesis, which although measured in 5 dpf larvae was labeled by EdU pulse at 24 hpf. Higuchi also found that treating developing embryos with 5 μM cortisol in the media from 0 to 5 dpf also decreased staining for markers of proliferation in 5 dpf forebrain.

Adding treatments directly to the media of developing embryos/larvae is an approach several laboratories have used. D’Agostino, et al., found that treatment with a high level of exogenous cortisol (100 μM) for the first 48 h post-fertilization increased the hatching rate of embryos. Expression level of *hsd11b2* (coding for the cortisol metabolizing HSD2 enzyme) was significantly and dynamically affected by the treatment as well, being ~7-fold higher than controls at 48 hpf, then decreasing to 1/3 of control levels at 72 hpf before rebounding back to greater than twofold controls at 96 hpf. Basal expression levels of the stress-responsive neuronal gene *c-fos* were increased by the treatment, while levels in response to physical (swirling) and osmotic challenges were, respectively, blunted and enhanced after treatment. In another study, treatment with 100 μM of the potent synthetic GC DEX for the first 5 days of development was found to suppress endogenous cortisol while also accelerating the hatch rate of embryos (Wilson et al., 2016). Conversely, knockdown of GR with morpholino was found to delay development and decrease larval activity and response to stimuli at 4 dpf. Larvae from both DEX and GR knockdown treatment groups were followed into adulthood, where fish treated with DEX during development were found to be larger and have elevated blood glucose and hepatic expression of the gluconeogenic gene *pepck*. DEX-treated adults also displayed more bold behavior and an increased cortisol response to stress, indicating long-term effects on HPI function of the developmental exposure. Interestingly, the authors also looked at developmental hypoxia as an experimental condition and found similar effects on larval development to those of GR knockdown. Intricate cross-regulation between hypoxia-inducible factor (HIF) and GC signaling pathways has recently been demonstrated using zebrafish larvae (Marchi et al., 2020), and stressors including HIF signaling have also been shown to regulate embryonic hematopoiesis *via* the HPI axis (Kwan et al., 2016).

Given the ease of adding treatments to water, zebrafish are increasingly used in screening for toxicant effects. In a screening of steroid hormones, modest decrease in activity level was found in larvae treated with GCs, along with a significant increase in trough expression level of circadian genes including *per1a* and *nr1d2a* (Zhao et al., 2018), two genes we have also found increased by chronic cortisol exposure (Gans et al., 2020, 2021). Another group found that environmentally relevant concentrations of cortisol and the synthetic prescription GC clobetasol propionate impacted larval muscle and heart function, as well as expression of genes involved in immunity, glucose metabolism, development, and the circadian clock (Willi et al., 2018). Interestingly, while clobetasol had larger effects on immune and developmental genes, cortisol had a greater effect on genes involved in glucose metabolism including *g6pca*, a gene we have found consistently elevated by chronic cortisol treatment as well (Gans et al., 2020). Effects of treatments on some genes were inconsistent when measured at different timepoints (96 or 120 hpf), or not significant until the later timepoint, perhaps highlighting the rapidly changing patterns of gene expression associated with development.

In the studies discussed above, a variety of synthetic and endogenous GCs over a range of dosage were utilized. We have focused on the effects of a chronic 1 μM dose of cortisol, to model a more physiologically relevant exposure. In 2016, Hartig et al. (2016) reported that this concentration was sufficient to significantly elevate GR receptor activity in larvae as measured *in vivo* by a fluorescent transgene reporter, and to elevate heart rate and staining for reactive oxygen species. RNA-sequencing (RNA-seq) of whole larvae at 5 dpf indicated that cortisol treatment from 0 to 5 dpf led to increased expression in genes involved largely in innate immunity and inflammation, as well as lipid catabolism, while genes involved in membrane polarization, synaptic transmission, and cell communication were under-expressed. Using an adult tail-fin injury model, it was found that although cortisol treatment ended at 5 dpf, effects on immune gene regulation were carried into adulthood (Hartig et al. 2016). Whereas control fish responded to injury by upregulating a collection of immune genes in the injured tissue at 2 days post-injury (dpi) and then resolved this response by 4dpi, tail fins of fish raised from treated embryos displayed generally higher basal expression of the same immune genes and a blunted or inverted dynamic response, which correlated with a higher percentage of defective tail fin regeneration. Similarly, the immune gene response to interperitoneally injected lipo-polysaccharide (to mimic bacterial infection) resulted in a blunted immune gene response in adults treated with chronic cortisol during early development.

Hartig et al. (2016) also found that chronic cortisol treatment during development caused increased whole-body cortisol in adulthood, indicating long-term effects on HPI output. In a follow-up experiment, evidence was found that cortisol production was increased in kidney tissue (the site of interrenal cells) of fish treated with developmental cortisol, and that in response to fasting stress more cortisol was taken up in target tissues including the brain (Hartig et al., 2020). Correspondingly, blood cortisol was higher than in controls in fed animals but lower than controls after fasting stress, seemingly indicating altered binding/transport kinetics. Unpublished preliminary data also indicate defective blood glucose homeostasis in these adult fish: whereas in control fish blood glucose levels are similar whether fed or fasted, fish exposed to elevated cortisol as embryos had depleted blood glucose after fasting, and elevated glucose after being re-fed (Gans, unpublished results). Together these data indicate that developmental exposure to elevated cortisol has long-term multisystemic effects, which involve changes in HPI axis dynamics and physiological processes governed by the axis including immunity, metabolism, and homeostasis.

To elucidate molecular mechanisms underlying these long-term physiological symptoms of developmental cortisol exposure, we performed an Assay for Transposase-Accessible Chromatin using Sequencing (ATAC-seq) on blood cells of adult fish (Hartig et al., 2020). This technique was used to assay whether chronic cortisol treatment from 0 to 5 dpf had long-term effects on chromatin accessibility. Among the loci where chromatin accessibility was most increased in treated animals were the promoters of several known GC targets including *klf9*, *sgk1, glcc1l*, and *fkbp5*. The promoter of *klf9* was the third-most affected site in the genome as well as the top-ranked transcription factor, and a further analysis of the ATAC-seq dataset showed that the binding motif for Klf9 was highly enriched in sequences from the top 250 peaks displaying more accessible chromatin. These data suggested that *klf9* may be not only a target of the GR, but a feed-forward regulator of the transcriptional response to GC. Among the most significantly affected genes in the ATAC-seq data *klf9, fkbp5*, and *chac1* (involved in redox and neurogenic pathways) were notable for also being among the most significantly upregulated genes in RNA-seq of treated 5 dpf larvae. Across several experiments using quantitative polymerase chain reactions (qPCR) to ascertain gene expression, levels of *klf9* and *fkbp5* were found to be highly correlated, and significantly increased by cortisol treatment overall, although not in every instance (Hartig et al., 2020).

## Krüppel-Like Factor 9: a Mediator of GC-Induced Developmental Programming?

Krüppel-Like Factor 9 (Klf9) is a member of the Krüppel-Like Factor (KLF) family of transcription factors, all of which share similarity to the transcriptional repressor Krüppel (German for “cripple”) first discovered in *Drosophila* for involvement in patterning the segmented larval body plan (Licht et al., 1990). In vertebrates, KLFs play important roles in development as well and are expressed dynamically throughout development in tissues derived from all three germ layers (endoderm, mesoderm and ectoderm; Bialkowska et al., 2017). Eighteen KLFs have been identified in the human genome, and 24 in the genome of zebrafish, with six human KLF (but not Klf9) having duplicate paralogs in *Danio* (Xue et al., 2015). All KLF members share a highly conserved DNA-binding domain (DBD) toward the C-terminus of the protein comprising three C2H2 zinc-finger domains. The DBD binds to G/C-rich sequence motifs common throughout the genome. The N-terminal ends of KLF proteins are more variable and contain domains for interacting with various transcriptional co-regulators. The N-terminus of Klf9 and its most closely related KLFs (“Group 3”) contains a domain for interacting with the Sin3a histone de-acetylation complex, while other KLF subgroups contain N-terminal domains known to interact with alternate co-regulators including acetyl-transferases and the repressive C-terminal Binding Protein (McConnell and Yang, 2010). Thus, regulation by KLFs may either promote or repress target gene expression, depending on the particular KLF as well as the cellular context and cofactor milieu. While both activating and repressive activities have been reported for Klf9 (Imataka et al., 1992; Mitchell and DiMario, 2010), recent evidence suggests that Klf9’s functional role can be predominantly repressive in a given tissue such as hippocampus (Knoedler et al., 2017). Given the variety of cofactors among different KLFs and their ability to bind similar genomic sequence motifs, it is believed that KLF proteins provide a context-sensitive level of fine-tuned transcriptional regulation; the precise regulation of target gene expression will depend upon which KLFs are expressed in a cell, as well as what upstream signals they are responsive too. KLFs respond to numerous signals including insulin and circulating hormones and are effectors of various nuclear hormone receptors, and it seems that they function as nodes to integrate and potentiate various inputs into a coordinated transcriptional response (Knoedler and Denver, 2014).

KLFs play important roles in basic cellular processes such as metabolism, where they contribute to matching the usage of macronutrients to energetic demands of the whole organism (Hsieh et al., 2018; Pollak et al., 2018). KLFs are also involved in determination of cell fate (Bialkowska et al., 2017). Klf4 was one of the four “Yamanaka Factors” first used to reprogram fibroblasts into pluripotent stem cells (Takahashi et al., 2007), and a core group of KLFs including Klf2, Klf4, and Klf5 was found necessary to maintain pluripotency of embryonic stem cells (Jiang et al., 2008). These KLFs are all members of a KLF subgroup (“Group 1”) defined by an N-terminal trans-activating domain. Loss of members of this group leads to embryonic or neonatal death in mice. Among these, *Klf2* and *Klf5* mutants exhibit defects in lung expansion (Wani et al., 1999) and surfactant production (Wan et al., 2008). In mouse macrophages *Klf2* has been shown to respond to DEX treatment (Chinenov et al., 2014). It is possible that a molecular pathway involving GCs and *Klf2* response could contribute to the enhanced fetal lung maturation caused by prenatal GC administration, but this possibility has not been tested to our knowledge. Interestingly, in mouse macrophages *Klf9* transcription was also induced by DEX, and the pulsatile *Klf2* response to DEX was lost when *Klf9* was knocked out, suggesting dynamic regulation of *Klf2* by an incoherent feedforward loop between the GR and Klf9 (Chinenov et al., 2014).

### Identification of Klf9 as a Direct GR Target

A body of evidence revealing that Klf9 is a direct target of the GR has come from the Denver Laboratory at the University of Michigan. However, studies first identified Klf9 (originally known as basic transcription element binding protein, BTEB or BTEB1) as a target of *thyroid* hormone (T3) in developing *Xenopus* and rat neurons (Denver et al., 1997, 1999). In rat neuronal cell culture, T3-induced expression of Klf9 only once cells became post-mitotic, and the regulation of Klf9 by T3 in brain was lost by postnatal day 30. This evidence suggests a limited window for regulation of Klf9 by T3. Notably this window overlaps with the stress hypo-responsive period in pups, during which inability to produce CORT in response to stress is thought to protect neurodevelopment (see previous sections). In confluent neuronal cell culture, forced overexpression of Klf9 caused an increase in neurite outgrowth, and in a related study Klf9 knock-down inhibited outgrowth (Cayrou and Denver, 2002). However, work by others has shown that Klf9 suppresses axon growth and regenerative capacity after nerve injury (Apara et al., 2017; Galvao et al., 2018). These seemingly disparate results are likely explained by differences in experimental designs/contexts, but they also exemplify our limited understanding of how complex regulatory networks can utilize the same basic transcription factor to generate nearly opposite physiological results.

Further experiments by the Denver laboratory revealed that acute stress also induces *Klf9* expression in *Xenopus* brain, mediated by cortisol and the GR. This response was not affected by inhibiting protein synthesis, suggesting Klf9 as an immediate target of the GR (Bonett et al., 2009). Chromatin immunoprecipitation using myc-tagged MR identified two putative MR/GR response elements (MRE/GRE) upstream of the *Klf9* TSS in HEK cells. In mouse hippocampal cells, these response elements were found to bind GR and promote transcription in reporter constructs upon treatment with cortisol, solidifying *Klf9* as a *bona fide* GC target (Bagamasbad et al., 2012). Both corticosteroid response elements were found conserved among Therian mammals, and one of the elements is more broadly conserved among all tetrapods including *Xenopus*. Intriguingly, the GRE conserved in tetrapods neighbors a thyroid hormone response element, and together these hormone response elements form a regulatory module capable of synergistically enhancing *Klf9* expression *via* combination of T3 and GC signals (Bagamasbad et al., 2015). Although the authors failed to identify either of the upstream GREs found in mammals in the genomes of zebrafish or other ray-finned fish, the *Danio* genomic sequence does in fact contain multiple putative GRE upstream of the *Klf9* TSS, including two found in approximately the same region as in tetrapods (roughly 5,500 bp and 3,500 bp upstream of the TSS, respectively). Using ATAC-seq, we found that chromatin around the *Danio klf9* −3,500 bp GRE was significantly more accessible in the blood of adult fish raised from cortisol-treated embryos as was chromatin around another predicted GRE located just 277 bp upstream from the TSS (Hartig et al., 2020). Although we did not identify an ATAC-seq peak at the −5,500 bp GRE, this location does contain a sequence motif that may be a potential thyroid hormone response element, which could perhaps be evidence of an ancestral ortholog to the tetrapod GC/thyroid hormone synergy module. While the lack of an ATAC-seq peak in our blood assay at −5,500 bp seems to argue against this, it could be also be simply due to tissue specificity, developmental stage, or another context. A recent study has shown that GC activation of *Klf9* expression in lung cells involves both proximal and distal GR-binding enhancer elements, combining constitutive enhancer-RNA production at proximal elements and rapidly inducible looping with distal enhancers (Mostafa et al., 2021).

### Evidence That Klf9 Is a Key Regulator of Glucocorticoid Signaling

Other laboratories have also found evidence of Klf9 involvement in GC signaling. Very recently, it was shown that *Klf9* induction by synthetic GCs in mouse liver leads to hyperglycemia, while deletion of *Klf9* caused hypoglycemia and was protective in a model of GC-induced diabetes (Cui et al., 2019). In adenocarcinoma lung cell culture DEX was found to induce expression of several KLFs, including *Klf9* (Reddy et al., 2009). Interestingly, in this study timing of GR-target gene expression generally differed with the direction of regulation: induction of GR-target gene expression happened significantly faster and in advance of repression of other GR targets. These dynamics are consistent with repressive feedforward regulatory logic playing a broad role in GC-dependent gene regulation, wherein increase of certain GR-targets could lead directly to subsequent repression of additional GC targets. An example of one such incoherent feed-forward loop circuit involving regulation of *Klf2* by the GR and KLF9 has been suggested by transcriptional dynamics and GR binding sites assayed in macrophages (Chinenov et al., 2014). Direct interaction between KLF9 and *Klf2* was not tested, however. In human keratinocytes, *Klf9* expression has been shown to be circadian, induced by GCs, and have an antiproliferative effect (Spörl et al., 2012). Also in keratinocytes, *positive* feed-forward regulation of GR activity by KLF9 was suggested by decreased GRE-luciferase reporter activity after KLF9 knockdown (Lili et al., 2019).

A recent meta-analysis of 17 transcriptomic studies investigating effects of GCs in brain or central nervous system-derived cell lines found that *Klf9* was the third-most consistently induced target of GC treatment, being significantly upregulated in seven of the studies (Juszczak and Stankiewicz, 2017). Notably *Klf9* expression was more consistently affected by GCs than some very well-known GC targets including *Fkbp5*. *Klf9* was also *the most* consistently affected transcription factor. Interestingly, though 9,605 genes were reported significantly affected by GC treatment in at least one of the studies analyzed, only 88 of these genes (0.9%) showed consistent up- or down-regulation in at least four of the studies, and only two genes in more than half of the studies.

**Figure 5:**
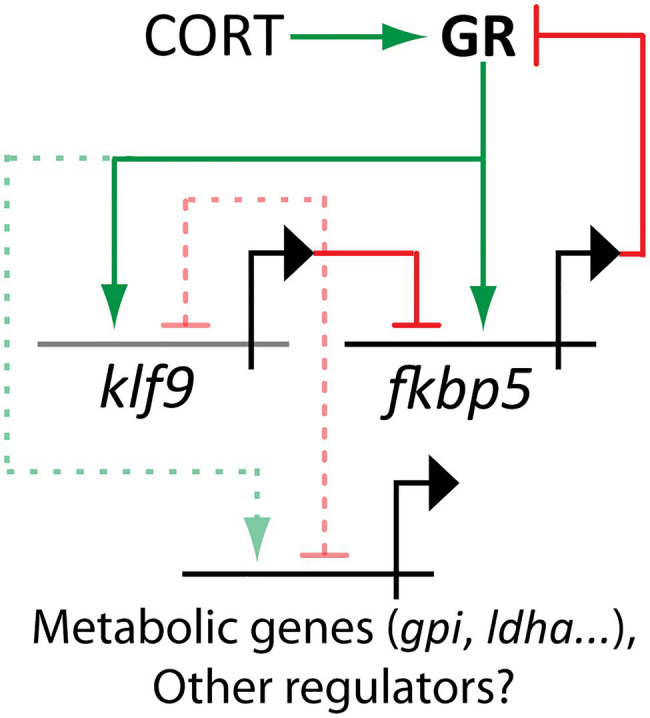
Klf9 is a node in the glucocorticoid-responsive gene regulatory network mediating feedforward control of glucocorticoid responsivity (*via fkbp5*) and metabolism. Activating interactions are depicted as arrows, inhibitory interactions as bars. Solid lines indicate established interactions, dashed lines are hypothesized (Gans et al., 2021 and unpublished data).

As mentioned above, we found that *klf9* was the transcriptional regulatory gene most strongly affected by developmental cortisol exposure, both in the exposed larvae (by RNA-seq) and in blood cells of adults (by ATAC-seq) obtained from those larvae (Hartig et al., 2016, 2020). Moreover, motif enrichment analysis of the ATAC-seq peaks scoring highest for differential accessibility in response to developmental cortisol exposure revealed significant enrichment for consensus KLF binding motifs, including for Klf9 (Hartig et al., 2020). To further interrogate the role of Klf9 in GC signaling, we used CRISPR both to create a *klf9* knockout in zebrafish (Gans et al., 2020), and to introduce a C-terminal epitope tag into the endogenous locus (Gans et al., 2021). Experiments with the knockout line revealed that the defensive/pro-inflammatory transcriptomic response to chronic cortisol that we had previously reported in wildtype larvae was absent in *klf9*^−/−^ mutants (Gans et al., 2020), which in contrast responded by upregulating genes involved in glycolysis and related metabolic pathways (Gans et al., 2021). Furthermore, we found that *klf9* expression is both oscillatory and synchronous with that of *fkpb5*, which appears to be directly repressed by Klf9 (Gans et al., 2021). Together these results suggest that *klf9* is a key node in the gene regulatory network regulating GC responsivity (Figure 5), both by co-regulating *fkbp5* in an incoherent feedforward loop with the GR, and by repressing glycolytic gene expression (possibly also *via* incoherent feedforward regulation) in a way that is predicted to favor flux through the pentose phosphate pathway (Gans et al., 2021). Incoherent feedforward regulation was previously shown to be a hallmark of GC signaling (Chen et al., 2013) and is a ubiquitous circuit motif underlying dynamic control relevant to the foregoing discussion, including pulse-generation, response acceleration, fold-change detection, oscillatory signal processing (pules counting), and biochemical/physiological adaptation (Mangan and Alon, 2003; Goentoro et al., 2009; Ma et al., 2009; Zhang et al., 2016; Adler et al., 2017; Alon, 2019). This, together with the emerging importance of Klf9 in circadian regulation, stress responsivity, metabolic plasticity, immune regulation, and neurogenesis (Bonett et al., 2009; Spörl et al., 2012; Chinenov et al., 2014; Besnard et al., 2018; Cui et al., 2019; Potashkin et al., 2019; Yu et al., 2019; Fan et al., 2020; Knoedler et al., 2020, 2021) indicate that further study of the role of KLF9 in stress-induced developmental programming is warranted.

## Summary and Conclusion

In this review, we have described the human health costs of chronic stress and associated allostatic load, how those relate to HPA axis function and GC signaling dynamics, and how the latter are affected by developmental programming that occurs in response to early life stress and cortisol exposure. Epidemiology shows that such exposures can have lifelong and even intergenerational consequences. However, such studies have struggled to reach a consensus, due primarily to lack of experimental control and/or resolution. Animal models, including both mammalian and zebrafish, have increased our understanding of the molecular mechanisms by which GCs exert their effects but have also illuminated complex dependencies of those effects on contextual factors such as tissue-type, dosage, and timing in terms of both exposure and life stage. The GR and its negative feedback regulator FKPB5 have well-established roles in HPA axis regulation and GC signaling, as well as in stress- and/or GC-induced developmental programming. More recently, the GR target and transcriptional regulatory gene *KLF9*, known to play critical roles in development and plasticity in multiple tissues including neurons, hepatocytes, keratinocytes, and macrophages, has emerged as a key feedforward regulator of cellular responsivity to GCs, likely in part *via* its roles as a regulator of *fkbp5* and metabolism. Future studies aimed at mapping the GC-responsive gene regulatory network and assessing how it functions in the context of development and physiology in different cell and tissue-types are needed to further elucidate the mechanisms underlying stress-induced developmental programming.

## Author Contributions

IG: literature review and initial draft. JC: editing, additional text and references, and figures. All authors contributed to the article and approved the submitted version.

## Funding

This work was supported by the MDI Biological Laboratory and grants from the National Institutes of Health (R03-HD099468, P20-GM104318, and P20-GM103423).

## Conflict of Interest

The authors declare that the research was conducted in the absence of any commercial or financial relationships that could be construed as a potential conflict of interest.

## Publisher’s Note

All claims expressed in this article are solely those of the authors and do not necessarily represent those of their affiliated organizations, or those of the publisher, the editors and the reviewers. Any product that may be evaluated in this article, or claim that may be made by its manufacturer, is not guaranteed or endorsed by the publisher.
